# Within- and Between-Individual Variation in Energy and Nutrient Intake in Japanese Adults: Effect of Age and Sex Differences on Group Size and Number of Records Required for Adequate Dietary Assessment

**DOI:** 10.2188/jea.JE20120106

**Published:** 2013-05-05

**Authors:** Azusa Fukumoto, Keiko Asakura, Kentaro Murakami, Satoshi Sasaki, Hitomi Okubo, Naoko Hirota, Akiko Notsu, Hidemi Todoriki, Ayako Miura, Mitsuru Fukui, Chigusa Date

**Affiliations:** 1 Department of Social and Preventive Epidemiology, Graduate School of Medicine, the University of Tokyo, Tokyo, Japan; 1 東京大学大学院 医学系研究科 社会予防疫学分野; 2 Department of Preventive Medicine and Public Health, Keio University School of Medicine, Tokyo, Japan; 2 慶應義塾大学 医学部 衛生学公衆衛生学教室; 3 Department of Social and Preventive Epidemiology, School of Public Health, the University of Tokyo, Tokyo, Japan; 3 東京大学大学院 医学系研究科 公共健康医学専攻 社会予防疫学分野; 4 Department of Health and Nutritional Science, Faculty of Human Health Science, Matsumoto University, Matsumoto, Nagano, Japan; 4 松本大学 人間健康学部 健康栄養学科; 5 Department of Food Science and Nutrition, Tottori College, Tottori, Japan; 5 鳥取短期大学 生活学科 食物栄養専攻; 6 Department of Public Health and Hygiene, School of Medicine, University of the Ryukyus, Nakagami-gun, Okinawa, Japan; 6 琉球大学大学院 医学研究科 衛生学・公衆衛生学講座; 7 Department of Health and Nutritional Science, Faculty of Health Promotional Science, Hamamatsu University, Hamamatsu, Shizuoka, Japan; 7 浜松大学 健康プロデュース学部 健康栄養学科; 8 Department of Statistics, Osaka City University Medical School, Osaka, Japan; 8 大阪市立大学 医学部 推計学研究室; 9 Department of Food Science and Nutrition, School of Human Science and Environment, University of Hyogo, Himeji, Hyogo, Japan; 9 兵庫県立大学 環境人間学部 公衆栄養学研究室

**Keywords:** nutrients, within- and between-individual variation, age, sex, Japanese

## Abstract

**Background:**

Information on within- and between-individual variation in energy and nutrient intake is critical for precisely estimating usual dietary intake; however, data from Japanese populations are limited.

**Methods:**

We used dietary records to examine within- and between-individual variation by age and sex in the intake of energy and 31 selected nutrients among Japanese adults. We also calculated the group size required to estimate mean intake for a group and number of days required both to rank individuals within a group and to assess an individual’s usual intake, all with appropriate arbitrary precision. A group of Japanese women (younger: 30–49 years, *n* = 58; older: 50–69 years, *n* = 63) and men (younger: 30–49 years, *n* = 54; older: 50–76 years, *n* = 67) completed dietary records for 4 nonconsecutive days in each season (16 days in total).

**Results:**

Coefficients of within-individual variation and between-individual variation were generally larger in the younger group than in the older group and in men as compared with women. The group size required to estimate a group’s mean intake, and number of days required to assess an individual’s usual intake, were generally larger for the younger group and for men. In general, a longer period was required to rank women and older adults.

**Conclusions:**

In a group of Japanese adults, coefficients of within-individual variation and between-individual variation, which were used to estimate the group size and number of records required for adequate dietary assessment, differed by age, sex, and nutrient.

## Abstract (ja)

**背景：:**

良好な精度をもって習慣的な食事摂取量を推定しようとする際、エネルギーや栄養素摂取量の個人内・個人間変動に関する情報は重要である。しかしながら、日本人における知見は限られている。

**方法：:**

日本人成人のエネルギーと31種類の栄養素摂取量の個人内・個人間変動を年齢別、性別に調査した。そして一定の精度の条件下で、集団の平均摂取量を推定するために必要な調査人数を算出した。また、集団内での個人の順位付けを目的とした調査および、個人の習慣的な摂取量の推定を目的とした調査に必要な日数を各々算出した。参加者は、日本人女性（若年群：30～49歳の58人、高齢群：50～69歳の63人）と男性（若年群：30～49歳の54人、高齢群：50～76歳の67人）であった。食事調査は食事記録法を用い、非連続の4日間の調査を季節ごとに実施した（合計16日間）。

**結果：:**

殆どの個人内・個人間変動係数は、若年群の方が高齢群よりも大きく、男性の方が女性よりも大きかった。そして、集団の平均摂取量を推定する際に必要な調査人数は、全般的に若年群と男性の方が多かった。また必要な調査日数については、概して個人の習慣的な摂取量の推定を目的とした場合は若年群と男性の方が長かったが、集団内での個人の順位付けが目的の場合は、高齢群と女性の方が長かった。

**結論：:**

日本人成人において、適切な食事調査に必要な調査人数と日数を検討する際に用いられる個人内・個人間変動係数は、年齢や性、栄養素によって異なることが示された。

## INTRODUCTION

Fluctuations in daily dietary intake values, which frequently hamper analysis of nutritional data, result from within- and between-individual variation.^1^^–^^3^ Within-individual variation is subject to several factors such as true day-to-day variation, variation by day of the week and season, and residual variation, including measurement error. Between-individual variation is strongly influenced by factors such as age and sex.^1^^–^^8^

These variations should be considered whenever dietary intake is assessed in individuals and groups.^3^^,^^9^ Properly designed nutritional research that includes dietary assessment should thus consider the number of subjects required in 1 group (group size) and the number of days required to implement the assessment efficiently.^3^^,^^10^ These variables can be estimated using within- and between-individual variation of nutrient intake.^1^^–^^3^^,^^7^ Dietary assessment is usually conducted for 1 of 3 purposes: (1) to compare the mean intake of different groups, (2) to rank individuals within a group, or (3) to assess an individual’s usual intake. Thus, knowledge of within- and between-individual variation is required in order to determine group size in studies comparing mean intake between groups,^7^ and the ratio of within- to between-individual variation is required in order to determine the number of days required for dietary assessment in studies that assess diet–disease associations using rankings of subjects within a group (eg, in estimating relative risk using quartile categorizations).^1^^,^^5^^,^^11^ Moreover, within-individual variation influences the number of days required to assess the usual intake of individuals (eg, to establish the true nature of dose-response).^1^^,^^3^^,^^9^

The magnitude of within- and between-individual variation in nutrient intake is largely determined by cultural and ecologic factors.^2^^,^^3^^,^^12^ The group size and number of days required for precise estimation of usual nutrient intake has been studied, but results have differed,^7^^,^^13^ and these variables might differ by age, sex, and country, due to different dietary habits.^13^ However, investigation of these issues has been limited in Japan.^4^^,^^14^^,^^15^

Here, we examined within- and between-individual variation in dietary intake by age and sex among Japanese adults. We assessed energy and 31 selected nutrients derived from dietary records (DRs) that were maintained for 4 nonconsecutive days in each season (16 days in total). We also estimated the group size required to estimate a group’s mean intake and the number of days required to rank individuals within a group and to assess an individual’s usual intake with adequate precision.

## METHODS

### Subjects

The study was conducted in 4 areas in Japan that differed in geographic conditions and dietary habits, namely Osaka (Osaka City: 11 743 persons/km^2^; urban), Nagano (Matsumoto City: 786 persons/km^2^; rural inland), Tottori (Kurayoshi City: 285 persons/km^2^; rural coastal), and Okinawa (Ginowan City: 4446 persons/km^2^; urban island),^16^ between November 2002 and September 2003.^17^^–^^20^ We recruited apparently healthy women aged 30 to 69 years who were willing to participate with a cohabiting husband. The subjects were volunteers and were asked by local staff to participate in the study. Subject recruitment was continued until a sufficient number of participants was obtained. In each of the 4 areas, each 10-year age band (30–39, 40–49, 50–59, and 60–69 years) included 8 women; the age of the husband was not considered. Thus, a total of 128 women and 128 men were invited. Dietitians were excluded from the study. None of the subjects had recently received dietary counseling from a doctor or dietitian or had a history of educational hospitalization for diabetes or nutritional education from a dietitian. Before the study, group orientations were held to explain the study purpose and design. Written informed consent was obtained from each subject. The study did not undergo ethical approval because it was conducted before ethical guidelines for epidemiologic research were enforced in Japan. However, use of data from this study was approved by the Ethics Committee at the University of Tokyo Faculty of Medicine (No. 3421). A total of 121 women aged 30 to 69 years and 121 men aged 30 to 76 years completed 16-day DRs and were included in the present analysis.

### Four 4-day semi-weighed dietary records

Between November 2002 and September 2003, each subject completed one 4-nonconsecutive-day semi-weighed DR in each of the 4 seasons at intervals of approximately 3 months: DR1 in November/December 2002 (autumn), DR2 in February 2003 (winter), DR3 in May 2003 (spring), and DR4 in August/September 2003 (summer).^17^^–^^20^ The 4 recording days consisted of 3 randomly selected weekdays and 1 weekend day. During the orientation session, local staff (registered dietitians) gave subjects both written and verbal instructions on how to keep the dietary record, using a completed recording sheet as an example. Each couple was given blank recording sheets and a digital scale (Tanita KD-173, ±2 g precision for 0–250 g and ±4 g precision for 251–1000 g). Subjects were also instructed on how to weigh each food item and drink and were asked to record and weigh all foods and drinks consumed on each recording day. When weighing was difficult (eg, when eating out), we instructed them to record the size and quantity of foods they ate as precisely as possible, using household measures. For each recording day, the subjects were asked to fax the completed forms to the local staff. The staff reviewed the submitted forms and, if necessary, asked the subject to augment and/or modify records by telephone or fax. The responses were faxed or, in some cases, handed directly to the staff.

All collected records were checked by trained registered dietitians in each local center and then again in the data center. The coding of records and conversion of measurements into grams were performed by trained registered dietitians in the survey center in accordance with uniform procedures. A total of 1398 food and beverage items appeared in the dietary records. Intake of energy and 31 selected nutrients was assessed based on the estimated intake of all items and the *Standard Tables of Food Composition in Japan*.^21^

### Anthropometric measurements, physical activity level, and reporting adequacy of reported energy intake

Body height and weight were measured to the nearest 0.1 cm and 0.1 kg, respectively, with subjects wearing light clothing and no shoes. Body mass index (BMI) was calculated as body weight (kg) divided by the square of body height (m). Basal metabolic rate (BMR) was calculated for each subject from age, measured body height, and weight with the use of the equations of Ganpule et al.^22^ Physical activity level (PAL) was obtained from a questionnaire that queried subjects on their occupation and leisure-time activity. PAL was classified into 1 of 4 categories, and the categorical classification of PAL was then converted to 1.5 for sedentary or light, 1.75 for active or moderate, and 2.0 for vigorous and heavy PAL (Ministry of Health, Labour and Welfare of Japan, 2009).^23^ Estimated energy requirement (EER) was calculated as the product of PAL and BMR. We used the ratio of reported energy intake (EI) to EER (EI/EER) as an indicator of the adequacy of energy intake reporting and defined a ratio of 1.0 as adequate reporting for the group.

### Statistical analysis

All statistical analyses were performed separately for women and men in 2 age groups (younger: 30–49 years for both women and men; older: 50–69 years for women and 50–76 years for men) using SAS statistical software, version 9.2 (SAS Institute Inc., Cary, NC, USA). Means, coefficients of within-individual variation (CV_w_) and between-individual variation (CV_b_), variance ratio, required group size, and required number of days were compared between age groups and sexes.

Means, SD, CV_w_, and CV_b_ for intakes were calculated. Variances of intake were estimated into 2 sources by 1-way ANOVA: (1) between-individual variance (σ_b_^2^) and (2) within-individual variance (σ_w_^2^) (ie, day-to-day variation unaccounted for by other sources). Estimates of σ_w_^2^ and σ_b_^2^ were calculated by setting mean squares equal to their expected values.

We used untransformed data to analyze within- and between-individual variation in energy and all nutrients because a previous study showed that the estimated relative contribution of sources of variance was not considerably affected by logarithmic transformation^2^ and because other previous studies showed that a logarithm and Box–Cox transformation did not improve the assumption of homoscedasticity across covariates in the models, that estimates based upon transformed nutrient data were difficult to interpret meaningfully, and that back-transformation would introduce bias to variance estimates.^24^^,^^25^

The group size of DR (G) required to estimate mean intakes with 95% CIs within the specified percentage deviation (D_0_) of group mean from group usual (“true”) mean intake was calculated using the following formula^2^: G = 1.96^2^ × [(CV_b_^2^ + CV_w_^2^)/D_0_^2^].

The number of days of DR (N_R_) required to ensure a specified level of correlation coefficient (r) between observed and unobserved usual (“true”) mean intakes in individuals was calculated using the following formula^1^^,^^7^: N_R_ = [r^2^/(1 − r^2^)] × VR, where VR is the variance ratio as determined by σ_w_^2^/σ_b_^2^. For this analysis, r is thus a measure of confidence of ranking or classification of individuals into fractions (eg, fourths).

The number of days of DR (N_I_) required to estimate mean intakes with 95% CIs within the specified percentage deviation (D_1_) of individual mean from usual (“true”) mean intake based on CV_w_ was calculated using the following formula^1^^–^^3^: N_I_ = (1.96 × CV_w_/D_1_)^2^.

## RESULTS

Table 1 shows the physical characteristics of men and women in the 2 age groups. The mean value of EI/EER was around 1.0 in all groups; the smallest value, 0.94, was for younger men, and largest value, 1.08, was for older women.

**Table 1.** Characteristics of study subjects according to sex and age group

	Women (*n* = 121)				Men (*n* = 121)			
	
	Younger^a^ (*n* = 58)		Older^a^ (*n* = 63)		Younger^a^ (*n* = 54)		Older^a^ (*n* = 67)	
			
	Mean	SD	Mean	SD	Mean	SD	Mean	SD
Age (years)	39.0	5.0	58.9	5.7	40.5	5.2	61.5	6.5
Body height (cm)	156.6	5.7	152.8	6.1	170.3	6.1	165.1	6.0
Body weight (kg)	52.9	6.9	53.8	7.2	67.9	11.1	65.2	9.6
BMI (kg/m^2^)	21.6	2.8	23.0	2.7	23.4	3.2	23.8	2.7
BMR (kcal/day)	1122	92	1046	111	1498	151	1368	145
Physical activity level	1.67	0.13	1.65	0.13	1.73	0.22	1.68	0.17
EI/EER	0.97	0.15	1.08	0.18	0.94	0.21	1.03	0.18

Abbreviations: BMI = body mass index; BMR = basal metabolic rate; EI = energy intake; EER = estimated energy requirement.

^a^Younger: 30–49 years for women and men; older: 50–69 years for women and 50–76 years for men.

Table 2 shows means, SD, CV_w_, CV_b_, and VR of daily intake of energy and 31 selected nutrients. Mean intake was larger in the older than in the younger group in both sexes for most nutrients and larger in men than in women for energy and all nutrients. CV_w_ was larger than CV_b_ for energy and most nutrients irrespective of age or sex. CV_w_ was larger in the younger than in the older group for both women (for energy and 26 nutrients; ±1%–65% differences) and men (for energy and 28 nutrients; ±2%–25% differences). The findings for CV_b_ were similar among both women (for energy and 26 nutrients; ±5%–12% differences) and men (for energy and 29 nutrients; ±8%–11% differences). Additionally, CV_w_ was larger in men than in women for both the younger (for energy and 21 nutrients; ±8%–4% differences) and older groups (for energy and 22 nutrients; ±7%–51% differences). Similar findings were obtained in CV_b_ for both the younger (for energy and 29 nutrients; ±1%–8% differences) and older groups (for energy and 18 nutrients; ±4%–8% differences). VR was greater than 1 for all except water (in younger women and men and older men) and carbohydrate (in younger men). In contrast to the results for CV_w_ and CV_b_, VR was larger in the older than in the younger group for both women (for energy and 21 nutrients) and men (for energy and 26 nutrients) and larger in women than in men for both the younger (for energy and 27 nutrients) and older groups (for energy and 16 nutrients).

**Table 2.** Mean daily energy and nutrient intake, coefficients of variation, and within- to between-individual variance ratios according to sex and age group

	Women (*n* = 121)										Men (*n* = 121)									
	
		Younger^a^ (*n* = 58)					Older^a^ (*n* = 63)					Younger^a^ (*n* = 54)					Older^a^ (*n* = 67)				
			
	Mean	SD	CV_w_ (%)^b^	CV_b_ (%)^c^	VR^d^	Mean	SD	CV_w_ (%)^b^	CV_b_ (%)^c^	VR^d^	Mean	SD	CV_w_ (%)^b^	CV_b_ (%)^c^	VR^d^	Mean	SD	CV_w_ (%)^b^	CV_b_ (%)^c^	VR^d^
Energy (kcal)	1824	327	20.6	17.2	1.44	1845	246	18.3	12.5	2.15	2392	473	21.1	19.0	1.23	2330	370	18.5	15.2	1.49
Protein (g)	65.1	11.6	25.5	16.6	2.37	72.9	10.6	23.5	13.4	3.08	81.0	16.9	25.4	19.8	1.64	86.8	13.6	23.7	14.5	2.67
Fat (g)	59.7	12.6	35.0	19.3	3.28	54.6	9.4	34.9	15.0	5.43	71.6	18.2	37.0	23.6	2.45	63.1	12.3	35.9	17.3	4.30
Carbohydrate (g)	244	51	20.6	20.4	1.02	258	41	18.5	15.1	1.50	311	69	20.9	21.6	0.93	312	52	19.9	15.9	1.57
Dietary fiber (g)	12.4	3.2	33.8	24.8	1.86	16.8	3.9	32.4	21.8	2.22	13.3	3.7	34.1	26.5	1.65	17.4	4.1	30.5	22.1	1.90
Water (g)	1902	403	20.6	20.6	1.00	2161	483	17.0	22.0	0.60	2356	615	23.3	25.5	0.84	2476	498	18.6	19.6	0.90
Sodium (mg)	3742	734	33.7	17.7	3.61	4315	780	34.4	15.9	4.67	4574	1008	35.7	20.2	3.13	5053	860	34.1	14.7	5.35
Potassium (mg)	2322	519	27.4	21.3	1.66	2994	548	26.7	17.0	2.46	2676	661	26.0	23.8	1.19	3207	571	23.9	16.8	2.03
Calcium (mg)	507	152	38.8	28.3	1.88	628	164	34.3	24.7	1.93	534	196	40.0	35.4	1.28	637	166	34.7	24.6	2.00
Magnesium (mg)	240	48	28.4	18.7	2.31	306	56	26.6	17.1	2.41	286	67	27.0	22.4	1.45	343	62	25.6	17.0	2.28
Phosphorus (mg)	983	197	24.6	19.1	1.65	1138	192	22.4	15.9	1.98	1187	275	24.0	22.4	1.15	1313	219	22.7	15.7	2.10
Iron (mg)	7.2	1.4	35.1	17.4	4.07	9.2	2.0	33.1	20.4	2.62	8.4	1.9	35.1	21.3	2.71	10.1	1.8	31.3	16.2	3.74
Zinc (mg)	7.7	1.5	31.4	17.6	3.19	8.3	1.3	28.1	13.6	4.28	9.8	2.2	32.4	21.2	2.34	10.0	1.6	30.3	13.8	4.86
β-carotene equivalent^e^ (µg)	2891	1036	84.4	29.0	8.48	4345	1334	62.0	26.5	5.48	3252	1130	80.0	28.4	7.91	4475	1377	65.9	26.0	6.44
Vitamin A^f^ (µg RE)	608	402	223.9	35.2	40.49	702	324	158.6	23.7	44.87	648	450	221.9	41.9	28.02	827	504	209.4	31.2	45.08
Vitamin D (µg)	6.0	2.2	105.6	25.3	17.38	9.4	3.7	99.9	30.6	10.66	7.4	2.7	106.0	24.4	18.82	11.3	4.5	93.3	32.0	8.52
α-tocopherol (mg)	6.9	1.5	36.5	20.1	3.30	7.9	1.5	36.9	16.3	5.12	8.0	2.0	39.9	23.0	3.01	8.8	1.8	38.1	17.7	4.65
Vitamin K (µg)	203	75	68.7	32.7	4.43	269	90	57.0	30.4	3.51	215	78	60.7	32.8	3.43	275	88	63.0	27.9	5.12
Vitamin B_1_ (mg)	0.8	0.2	41.2	17.8	5.32	0.9	0.2	34.1	14.3	5.71	1.0	0.2	44.9	21.0	4.57	1.1	0.2	36.5	14.6	6.30
Vitamin B_2_ (mg)	1.2	0.3	38.1	20.2	3.55	1.4	0.3	28.9	19.2	2.26	1.4	0.4	36.3	24.2	2.26	1.6	0.3	33.0	17.4	3.59
Niacin (mg)	15.9	3.6	38.5	20.4	3.57	18.3	3.7	34.7	18.3	3.58	21.6	5.8	39.4	24.8	2.51	22.6	5.6	36.4	23.2	2.47
Vitamin B_6_ (mg)	1.1	0.2	33.4	20.0	2.78	1.4	0.3	28.6	17.2	2.76	1.4	0.4	34.9	24.8	1.97	1.6	0.3	30.0	18.8	2.55
Vitamin B_12_ (µg)	6.4	2.6	103.8	30.3	11.73	8.7	3.0	88.6	26.0	11.63	8.0	3.6	96.1	38.5	6.23	10.9	4.2	96.4	29.7	10.54
Folate (µg)	300	82	51.8	24.0	4.67	411	97	39.1	21.4	3.33	339	96	53.6	25.0	4.58	451	103	49.6	19.2	6.69
Vitamin C (mg)	87.7	29.7	52.0	31.3	2.76	136.7	34.8	43.4	23.0	3.54	94.3	36.8	53.1	36.7	2.10	140.4	40.8	50.4	26.2	3.70
SFA (g)	17.3	4.3	40.9	22.6	3.28	15.1	3.2	40.8	18.8	4.71	20.2	6.4	45.1	29.7	2.31	16.9	3.5	41.3	18.2	5.16
MUFA (g)	21.6	5.0	40.7	20.8	3.85	18.8	3.7	41.2	17.0	5.90	26.6	7.0	42.5	24.2	3.09	22.3	5.3	42.4	21.1	4.02
PUFA (g)	12.9	2.4	40.3	15.9	6.42	12.8	2.3	40.1	14.9	7.21	15.9	3.5	40.7	19.2	4.47	14.8	3.0	39.7	17.8	5.00
n-6 PUFA (g)	10.7	2.1	42.0	16.2	6.69	10.2	1.9	43.3	14.9	8.45	13.0	2.9	42.8	19.5	4.80	11.7	2.5	42.6	18.6	5.26
n-3 PUFA (g)	2.2	0.5	55.9	20.0	7.82	2.6	0.6	57.1	19.0	9.02	2.8	0.7	57.0	22.3	6.51	3.1	0.8	57.8	21.2	7.47
Marine origin n-3 PUFA^g^ (mg)	687	289	119.5	29.6	16.32	1030	392	104.1	27.7	14.15	900	411	123.9	33.6	13.57	1312	524	99.0	31.4	9.94
Cholesterol (mg)	330	83	52.8	21.6	5.97	332	79	51.3	20.0	6.60	397	103	49.0	23.0	4.54	398	103	47.6	23.0	4.28

Abbreviations: CV_w_ = coefficient of within-individual variation; CV_b_ = coefficient of between-individual variation; VR = ratio of within- to between-individual variance; RE = retinol equivalents; SFA = saturated fatty acids; MUFA = monounsaturated fatty acids; PUFA = polyunsaturated fatty acids.

^a^Younger: 30–49 years for women and men; older: 50–69 years for women and 50–76 years for men.

^b^CV_w_ = [(within-individual variance)^0.5^/mean] × 100.

^c^CV_b_ = [(between-individual variance)^0.5^/mean] × 100.

^d^VR = within-individual/between-individual variance ratio (σ_w_^2^/σ_b_^2^).

^e^Sum of β-carotene, α-carotene/2, and cryptoxanthin/2.

^f^Sum of retinol, β-carotene/12, α-carotene/24, and cryptoxanthin/24.

^g^Sum of eicosapentaenoic acid, docosapentaenoic acid, and docosahexaenoic acid.

Table 3 shows the group size required to estimate mean intake of energy and nutrients with 95% CIs within a specified (ie, 2.5%, 5%, 10%, and 20%) deviation of a group’s mean from the group’s usual (“true”) mean intake by DR. The group size required to determine the mean intake of the group was larger in the younger than in the older group for both women (for energy and 29 nutrients) and men (for energy and 30 nutrients) and was larger in men than in women for both the younger (for energy and 26 nutrients) and the older groups (for energy and 22 nutrients).

**Table 3.** Group size required to estimate mean intake of energy and nutrients with 95% CIs within the specified % deviation (D_0_) of a group’s mean from the group’s usual (“true”) mean intake by dietary record according to sex and age group^a^

	Women (*n* = 121)								Men (*n* = 121)							
	
	Younger^b^ (*n* = 58)				Older^b^ (*n* = 63)				Younger^b^ (*n* = 54)				Older^b^ (*n* = 67)			
			
D_0_	2.5%	5%	10%	20%	2.5%	5%	10%	20%	2.5%	5%	10%	20%	2.5%	5%	10%	20%
Energy	442	111	28	7	302	76	19	5	497	124	31	8	353	88	22	6
Protein	569	142	36	9	448	112	28	7	639	160	40	10	476	119	30	7
Fat	980	245	61	15	884	221	55	14	1186	297	74	19	976	244	61	15
Carbohydrate	517	129	32	8	352	88	22	5	556	139	35	9	400	100	25	6
Dietary fiber	1081	270	68	17	937	234	59	15	1145	286	72	18	872	218	54	14
Water	520	130	32	8	473	118	30	7	732	183	46	11	448	112	28	7
Sodium	889	222	56	14	881	220	55	14	1032	258	64	16	846	212	53	13
Potassium	741	185	46	12	618	155	39	10	764	191	48	12	524	131	33	8
Calcium	1416	354	88	22	1096	274	69	17	1752	438	109	27	1110	278	69	17
Magnesium	712	178	44	11	614	154	38	10	757	189	47	12	580	145	36	9
Phosphorus	596	149	37	9	464	116	29	7	661	165	41	10	467	117	29	7
Iron	946	236	59	15	929	232	58	15	1038	260	65	16	763	191	48	12
Zinc	794	198	50	12	598	149	37	9	921	230	58	14	682	170	43	11
β-carotene equivalent^c^	4889	1222	306	76	2793	698	175	44	4426	1106	277	69	3085	771	193	48
Vitamin A^d^	31 569	7892	1973	493	15 808	3952	988	247	31 332	7833	1958	490	27 544	6886	1722	430
Vitamin D	7246	1812	453	113	6715	1679	420	105	7279	1820	455	114	5977	1494	374	93
α-tocopherol	1068	267	67	17	1002	250	63	16	1303	326	81	20	1085	271	68	17
Vitamin K	3558	890	222	56	2568	642	161	40	2925	731	183	46	2919	730	182	46
Vitamin B_1_	1237	309	77	19	842	210	53	13	1511	378	94	24	951	238	59	15
Vitamin B_2_	1141	285	71	18	738	184	46	12	1171	293	73	18	854	214	53	13
Niacin	1168	292	73	18	946	237	59	15	1331	333	83	21	1147	287	72	18
Vitamin B_6_	933	233	58	15	687	172	43	11	1127	282	70	18	770	193	48	12
Vitamin B_12_	7191	1798	449	112	5235	1309	327	82	6585	1646	412	103	6254	1563	391	98
Folate	2001	500	125	31	1219	305	76	19	2147	537	134	34	1741	435	109	27
Vitamin C	2261	565	141	35	1483	371	93	23	2564	641	160	40	1980	495	124	31
SFA	1344	336	84	21	1243	311	78	19	1789	447	112	28	1251	313	78	20
MUFA	1284	321	80	20	1222	305	76	19	1471	368	92	23	1378	344	86	22
PUFA	1155	289	72	18	1127	282	70	18	1245	311	78	19	1162	291	73	18
n-6 PUFA	1244	311	78	19	1290	323	81	20	1362	341	85	21	1326	332	83	21
n-3 PUFA	2170	543	136	34	2224	556	139	35	2301	575	144	36	2332	583	146	36
Marine origin n-3 PUFA^e^	9315	2329	582	146	7134	1784	446	111	10 124	2531	633	158	6624	1656	414	103
Cholesterol	2000	500	125	31	1862	465	116	29	1803	451	113	28	1715	429	107	27

Abbreviations: SFA = saturated fatty acids; MUFA = monounsaturated fatty acids; PUFA = polyunsaturated fatty acids.

^a^Group size of dietary record assuming single observation for each individual = 1.96^2^ × [(CV_b_^2^ + CV_w_^2^)/D_0_^2^], where D_0_ = the specified % deviation of group mean from group usual (“true”) mean intake.

^b^Younger: 30–49 years for women and men; older: 50–69 years for women and 50–76 years for men.

^c^Sum of β-carotene, α-carotene/2, and cryptoxanthin/2.

^d^Sum of retinol, β-carotene/12, α-carotene/24, and cryptoxanthin/24.

^e^Sum of eicosapentaenoic acid, docosapentaenoic acid, and docosahexaenoic acid.

Table 4 presents the number of days required to ensure specified (ie, 0.75, 0.80, 0.85, 0.90, and 0.95) correlation coefficients between observed and usual (“true”) mean intake of energy and nutrients by DR. The number of days required to rank individuals within a group by intake was larger in the older than in the younger group for both women (for energy and 20 nutrients) and men (for energy and 25 nutrients) and was larger in women than in men for both the younger (for energy and 29 nutrients) and older groups (for energy and 16 nutrients).

**Table 4.** Number of days required to ensure a specified correlation coefficient (r) between observed and usual (“true”) mean intake of energy and nutrients by dietary record according to sex and age group^a^

	Women (*n* = 121)										Men (*n* = 121)									
	
	Younger^b^ (*n* = 58)					Older^b^ (*n* = 63)					Younger^b^ (*n* = 54)					Older^b^ (*n* = 67)				
			
r	0.75	0.8	0.85	0.9	0.95	0.75	0.8	0.85	0.9	0.95	0.75	0.8	0.85	0.9	0.95	0.75	0.8	0.85	0.9	0.95
Energy	2	3	4	6	13	3	4	6	9	20	2	2	3	5	11	2	3	4	6	14
Protein	3	4	6	10	22	4	5	8	13	28	2	3	4	7	15	3	5	7	11	25
Fat	4	6	9	14	30	7	10	14	23	50	3	4	6	10	23	6	8	11	18	40
Carbohydrate	1	2	3	4	9	2	3	4	6	14	1	2	2	4	9	2	3	4	7	15
Dietary fiber	2	3	5	8	17	3	4	6	9	21	2	3	4	7	15	2	3	5	8	18
Water	1	2	3	4	9	1	1	2	3	6	1	1	2	4	8	1	2	2	4	8
Sodium	5	6	9	15	33	6	8	12	20	43	4	6	8	13	29	7	10	14	23	49
Potassium	2	3	4	7	15	3	4	6	10	23	2	2	3	5	11	3	4	5	9	19
Calcium	2	3	5	8	17	2	3	5	8	18	2	2	3	5	12	3	4	5	9	18
Magnesium	3	4	6	10	21	3	4	6	10	22	2	3	4	6	13	3	4	6	10	21
Phosphorus	2	3	4	7	15	3	4	5	8	18	1	2	3	5	11	3	4	5	9	19
Iron	5	7	11	17	38	3	5	7	11	24	3	5	7	12	25	5	7	10	16	35
Zinc	4	6	8	14	29	6	8	11	18	40	3	4	6	10	22	6	9	13	21	45
β-carotene equivalent^c^	11	15	22	36	79	7	10	14	23	51	10	14	21	34	73	8	11	17	27	60
Vitamin A^d^	52	72	105	173	375	58	80	117	191	415	36	50	73	119	259	58	80	117	192	417
Vitamin D	22	31	45	74	161	14	19	28	45	99	24	33	49	80	174	11	15	22	36	79
α-tocopherol	4	6	9	14	31	7	9	13	22	47	4	5	8	13	28	6	8	12	20	43
Vitamin K	6	8	12	19	41	5	6	9	15	32	4	6	9	15	32	7	9	13	22	47
Vitamin B_1_	7	9	14	23	49	7	10	15	24	53	6	8	12	19	42	8	11	16	27	58
Vitamin B_2_	5	6	9	15	33	3	4	6	10	21	3	4	6	10	21	5	6	9	15	33
Niacin	5	6	9	15	33	5	6	9	15	33	3	4	7	11	23	3	4	6	11	23
Vitamin B_6_	4	5	7	12	26	4	5	7	12	26	3	4	5	8	18	3	5	7	11	24
Vitamin B_12_	15	21	31	50	109	15	21	30	50	108	8	11	16	27	58	14	19	27	45	98
Folate	6	8	12	20	43	4	6	9	14	31	6	8	12	20	42	9	12	17	29	62
Vitamin C	4	5	7	12	26	5	6	9	15	33	3	4	5	9	19	5	7	10	16	34
SFA	4	6	9	14	30	6	8	12	20	44	3	4	6	10	21	7	9	13	22	48
MUFA	5	7	10	16	36	8	10	15	25	55	4	5	8	13	29	5	7	10	17	37
PUFA	8	11	17	27	59	9	13	19	31	67	6	8	12	19	41	6	9	13	21	46
n-6 PUFA	9	12	17	29	62	11	15	22	36	78	6	9	13	20	44	7	9	14	22	49
n-3 PUFA	10	14	20	33	72	12	16	23	38	83	8	12	17	28	60	10	13	19	32	69
Marine origin n-3 PUFA^e^	21	29	42	70	151	18	25	37	60	131	17	24	35	58	126	13	18	26	42	92
Cholesterol	8	11	16	25	55	8	12	17	28	61	6	8	12	19	42	6	8	11	18	40

Abbreviations: SFA = saturated fatty acids; MUFA = monounsaturated fatty acids; PUFA = polyunsaturated fatty acids.

^a^Number of days of dietary record = [r^2^/(1 − r^2^)] × VR, where r = unobservable correlation coefficient between observed and usual (“true”) mean intakes of individuals and VR = within-individual/between-individual variance ratio (σ_w_^2^/σ_b_^2^).

^b^Younger: 30–49 years for women and men; older: 50–69 years for women and 50–76 years for men.

^c^Sum of β-carotene, α-carotene/2, and cryptoxanthin/2.

^d^Sum of retinol, β-carotene/12, α-carotene/24, and cryptoxanthin/24.

^e^Sum of eicosapentaenoic acid, docosapentaenoic acid, and docosahexaenoic acid.

Table 5 shows the number of days required to assess mean intake of energy and nutrients with 95% CIs within a specified (ie, 5%, 10%, 20%, and 30%) deviation of an individual’s mean from usual (“true”) mean intake by DR. The number of days needed to assess the usual intake of individuals was larger in the younger than in the older group for both women (for energy and 26 nutrients) and men (for energy and 28 nutrients) and was larger in men than in women for both the younger (for energy and 20 nutrients) and older groups (for energy and 21 nutrients).

**Table 5.** Number of days required to assess mean intake of energy and nutrients with 95% CIs within the specified % deviation (D_1_) of an individual’s mean from usual (“true”) mean intake by dietary record according to sex and age group^a^

	Women (*n* = 121)								Men (*n* = 121)							
	
	Younger^b^ (*n* = 58)				Older^b^ (*n* = 63)				Younger^b^ (*n* = 54)				Older^b^ (*n* = 67)			
			
D_1_	5%	10%	20%	30%	5%	10%	20%	30%	5%	10%	20%	30%	5%	10%	20%	30%
Energy	65	16	4	2	52	13	3	1	69	17	4	2	53	13	3	1
Protein	100	25	6	3	85	21	5	2	99	25	6	3	87	22	5	2
Fat	188	47	12	5	187	47	12	5	211	53	13	6	198	49	12	5
Carbohydrate	65	16	4	2	53	13	3	1	67	17	4	2	61	15	4	2
Dietary fiber	176	44	11	5	161	40	10	4	178	45	11	5	143	36	9	4
Water	65	16	4	2	44	11	3	1	84	21	5	2	53	13	3	1
Sodium	174	44	11	5	181	45	11	5	195	49	12	5	178	45	11	5
Potassium	116	29	7	3	110	27	7	3	104	26	6	3	88	22	5	2
Calcium	231	58	14	6	181	45	11	5	246	61	15	7	185	46	12	5
Magnesium	124	31	8	3	109	27	7	3	112	28	7	3	101	25	6	3
Phosphorus	93	23	6	3	77	19	5	2	88	22	6	2	79	20	5	2
Iron	190	47	12	5	168	42	11	5	190	47	12	5	150	38	9	4
Zinc	151	38	9	4	121	30	8	3	161	40	10	4	141	35	9	4
β-carotene equivalent^c^	1093	273	68	30	591	148	37	16	982	246	61	27	667	167	42	19
Vitamin A^d^	7702	1926	481	214	3866	966	242	107	7563	1891	473	210	6737	1684	421	187
Vitamin D	1713	428	107	48	1535	384	96	43	1728	432	108	48	1337	334	84	37
α-tocopherol	205	51	13	6	210	52	13	6	245	61	15	7	223	56	14	6
Vitamin K	726	181	45	20	500	125	31	14	566	142	35	16	610	153	38	17
Vitamin B_1_	260	65	16	7	179	45	11	5	310	77	19	9	205	51	13	6
Vitamin B_2_	222	56	14	6	128	32	8	4	203	51	13	6	167	42	10	5
Niacin	228	57	14	6	185	46	12	5	238	60	15	7	204	51	13	6
Vitamin B_6_	172	43	11	5	126	32	8	4	187	47	12	5	138	35	9	4
Vitamin B_12_	1657	414	104	46	1205	301	75	33	1418	355	89	39	1428	357	89	40
Folate	412	103	26	11	234	59	15	7	441	110	28	12	379	95	24	11
Vitamin C	415	104	26	12	289	72	18	8	434	108	27	12	390	97	24	11
SFA	257	64	16	7	256	64	16	7	312	78	20	9	262	65	16	7
MUFA	255	64	16	7	261	65	16	7	278	69	17	8	276	69	17	8
PUFA	250	62	16	7	247	62	15	7	254	64	16	7	242	61	15	7
n-6 PUFA	271	68	17	8	288	72	18	8	282	70	18	8	279	70	17	8
n-3 PUFA	481	120	30	13	501	125	31	14	499	125	31	14	514	129	32	14
Marine origin n-3 PUFA^e^	2194	549	137	61	1666	416	104	46	2357	589	147	65	1505	376	94	42
Cholesterol	428	107	27	12	404	101	25	11	369	92	23	10	348	87	22	10

Abbreviations: SFA = saturated fatty acids; MUFA = monounsaturated fatty acids; PUFA = polyunsaturated fatty acids.

^a^Number of days of dietary record = (1.96 × CV_w_/D_1_)^2^, where D_1_ = the specified % deviation of individual mean from usual (“true”) mean intake.

^b^Younger: 30–49 years for women and men; older: 50–69 years for women and 50–76 years for men.

^c^Sum of β-carotene, α-carotene/2, and cryptoxanthin/2.

^d^Sum of retinol, β-carotene/12, α-carotene/24, and cryptoxanthin/24.

^e^Sum of eicosapentaenoic acid, docosapentaenoic acid, and docosahexaenoic acid.

## DISCUSSION

In this study of Japanese women and men, we found that coefficients of within-individual variation and between-individual variation were generally larger in the younger group than in the older group, whereas variance ratio was larger in the older group than in the younger group. Similarly, both CV_w_ and CV_b_ were generally larger in men than in women, whereas VR was larger in women than in men. To our knowledge, this study is the first to examine within- and between-individual variation in dietary intake with respect to both age and sex in a Japanese population and in Asian men.

The results of this study are comparable with those of previous studies in Japan,^4^^,^^14^^,^^15^ namely, CV_w_ was larger than CV_b_, and CV_w_, CV_b_, and VR were relatively small for energy, carbohydrate, protein, and water, intermediate for minerals, dietary fiber, and fat, and large for fatty acids, cholesterol, and vitamins. Ogawa et al used four 3-day DRs to investigate women (aged 47–76 years) and men (aged 45–77 years) living in a rural area.^15^ Their results for CV_w_ and CV_b_ were similar to our estimates. Egami et al used four 4-day DRs to assess women and men (aged >40 years) living in a coastal area.^14^ CV_w_ was generally larger than in our results, whereas CV_b_ was smaller. Tokudome et al used four 7-day DRs to investigate female dietitians (aged 32–66 years).^4^ CV_w_ and CV_b_ were generally smaller than in our study, possibly due to differences in eating patterns between their and our groups, which arose from the greater nutritional knowledge of their subjects.

Our findings are also consistent with those of several studies that examined CV_w_ and CV_b_ by age or sex.^7^^,^^15^^,^^26^ In a study of UK adults (a comparison among 4 groups categorized by sex and age, with younger groups aged 18–57 years using 7-day DRs vs older groups aged 60–80 years using three 7-day DRs), CV_w_ and CV_b_ were larger in the younger than in the older group for both sexes.^7^ In studies of Japanese adults living in a rural area (mentioned above),^15^ UK adults (mentioned above),^7^ and US elderly adults (aged >60 years using 3-day DRs),^26^ CV_w_ and CV_b_ were larger in men than in women. In a study of Chinese women (aged 40–59 years vs 60–70 years using 24-h dietary recall), while CV_w_ was consistent with our study, CV_b_ was larger in the older than in the younger group.^5^ Additionally, in studies of Japanese adults living in a coastal area (mentioned above),^14^ Korean elderly (mean [SD] age 70.4 [5.8] years using 5- or 6-day 24-h dietary recall),^12^ and Canadian adults (aged 25–44 years using 24-h dietary recall),^2^^,^^27^ CV_w_ and CV_b_ were larger in women than in men. These inconsistent results in some previous studies may be due to differences in study design: these studies^2^^,^^5^^,^^12^^,^^27^ used 24-h dietary recall, whereas we used DR. Cultural factors also likely played a role.^2^^,^^3^

The present results have implications for the design and interpretation of dietary assessment. First, among older adults and women, nutrient intake may be more homogeneous from day-to-day and among subjects than for younger adults and men, because smaller CV_w_ and CV_b_ were observed in older groups and in women than in their respective counterparts. Thus, as compared with men and younger adults, women and older adults may require a smaller group size and fewer days to assess the group’s and individual’s usual nutrient intake. Second, subjects can be more precisely ranked in groups of younger adults and/or men, because a smaller VR was observed in these groups. A smaller VR means that σ_w_^2^ is relatively small compared with σ_b_^2^ and that the difference in intake between individuals can be more easily distinguished. Therefore, if dietary assessment is conducted in individuals or groups by the same methods (number of days and group size) regardless of age or sex, the level of precision of the assessment will differ among the individuals or groups. If an analysis includes estimates of intake with a low level of precision, even in only 1 group, this may decrease the power of the statistical analysis and lead to misinterpretation of the association between dietary factors and an outcome.^2^^,^^7^^,^^9^^,^^12^ Third, regardless of age or sex, a large CV_w_ means that many DR days would be required to characterize an individual’s usual intake—for example, 4 to 481 days would be needed to achieve within 20% deviation for younger women. Therefore, use of an alternative method (eg, a semi-quantitative food frequency questionnaire) that can estimate usual intakes over a longer period than DR or dietary recall may be necessary to accord with the study objective, study design, demographic characteristics of the population, and available resources.^3^^,^^6^^,^^12^^,^^15^

Several limitations of this study warrant mention. First, the generalizability of our results is hampered by the fact that the present subjects were not randomly sampled from the general Japanese population but were instead volunteers and possibly health-conscious. As we lacked information on the subjects’ characteristics, including education and occupation, we could not determine how such characteristics influenced our findings. Mennen et al^13^ assumed that the dietary recall of subjects who completed a protocol is more precise (smaller CV_w_) than that of subjects who dropped out. Hebert et al^11^ suggested that CV_b_ is smaller in a population with higher socioeconomic status (SES). Thus, because of precise recording, CV_w_ might have been smaller in our volunteers than in the general population consuming a similar diet. CV_b_ might have been smaller because of limited variation in some variables (eg, health-consciousness). If so, the group size required to estimate a group’s mean intake in the general population would be larger than the estimates observed here (Table 3). Additionally, the number of days required to precisely estimate an individual’s usual intake in the general population would be larger than the estimates observed here (Table 5). Conversely, as we did not know whether VR was lower or higher in our volunteers than in the general population, the number of days required to rank individuals based on their intakes within the general population is unclear, that is, we cannot conclude that the required number of days is larger or smaller than the estimates observed here (Table 4).

Second, the subjects were married men and women living together, who likely frequently have the same meals. This implies that the CV_w_ and CV_b_ of men in this study might be underestimated as compared with the general male population because the daily menu is probably usually decided by women, who in our study had a smaller CV_w_ and CV_b_. Third, although we compared within- and between-individual variation between sexes and age groups (younger vs older), several unanticipated confounding factors, such as SES, might be present in our analysis. If the distribution of SES differs between sexes or age groups, and SES has an effect on dietary habits, it should be adjusted for in the analysis. However, we designed the study so as to consider important confounding factors that may affect the comparisons. For example, sex itself is an important confounding factor in a comparison between age groups, and age is the same in a comparison between sexes. To address this problem, we recruited the same number of subjects for each sex and age category. Living area, season, and timing of data collection (weekday or weekend day) are other possible confounding factors, and they were equalized between sexes and age groups.^1^^–^^4^^,^^28^^,^^29^ Finally, DR is susceptible to measurement error due to erroneous recording and potential changes in eating behavior.^3^ However, the adequacy of reported energy intake was likely adequate at the group level, given that the mean value of EI/EER was around 1.0.

In conclusion, the present study of Japanese adults showed that CV_w_ and CV_b_ were larger in a younger group than in an older group and larger in men than in women for energy and most nutrients. Precise estimation of usual nutrient intakes requires consideration of differences not only in CV_w_ and CV_b_ by age and sex, but also in group size and number of days estimated using CV_w_ and CV_b_. The present findings may have important implications for the design and interpretation of dietary assessment in Japanese adults.

## ONLINE ONLY MATERIALS

Abstract in Japanese.
