# Supplementary material for: Within- and Between-Individual Variation in Energy and Nutrient Intake in Japanese Adults: Effect of Age and Sex Differences on Group Size and Number of Records Required for Adequate Dietary Assessment
Source: J Epidemiol. 2013 May 5;23(3):178–86. doi: 10.2188/jea.JE20120106 (PMC3700253; doi:10.2188/jea.JE20120106)
Supplement: Abstract in Japanese. [file je-23-178-s001.pdf]

日本人成人におけるエネルギーと栄養素摂取量の個人内・個人間変動：適切な食事調査に必要な調査人数と日数に対する年齢と性の影響

福元　梓[1]、朝倉　敬子[2]、村上　健太郎[3]、佐々木　敏[3,*]、大久保　公美[1]、廣田　直子[4]、野津　あきこ[5]、等々力　英美[6]、三浦　綾子[7]、福井　充[8]、伊達　ちぐさ[9]

1 東京大学大学院　医学系研究科　社会予防疫学分野
2 慶應義塾大学　医学部　衛生学公衆衛生学教室
3 東京大学大学院　医学系研究科　公共健康医学専攻　社会予防疫学分野
4 松本大学　人間健康学部　健康栄養学科
5 鳥取短期大学　生活学科　食物栄養専攻
6 琉球大学大学院　医学研究科　衛生学・公衆衛生学講座
7 浜松大学　健康プロデュース学部　健康栄養学科
8 大阪市立大学　医学部　推計学研究室
9 兵庫県立大学　環境人間学部　公衆栄養学研究室

抄録

背景：良好な精度をもって習慣的な食事摂取量を推定しようとする際、エネルギーや栄養素摂取量の個人内・個人間変動に関する情報は重要である。しかしながら、日本人における知見は限られている。

方法：日本人成人のエネルギーと 31 種類の栄養素摂取量の個人内・個人間変動を年齢別、性別に調査した。そして一定の精度の条件下で、集団の平均摂取量を推定するために必要な調査人数を算出した。また、集団内での個人の順位付けを目的とした調査および、個人の習慣的な摂取量の推定を目的とした調査に必要な日数を各々算出した。参加者は、日本人女性（若年群：30～49 歳の 58 人、高齢群：50～69 歳の 63 人）と男性（若年群：30～49 歳の 54 人、高齢群：50～76 歳の 67 人）であった。食事調査は食事記録法を用い、非連続の 4 日間の調査を季節ごとに実施した（合計 16 日間）。

結果：殆どの個人内・個人間変動係数は、若年群の方が高齢群よりも大きく、男性の方が女性よりも大きかった。そして、集団の平均摂取量を推定する際に必要な調査人数は、全般的に若年群と男性の方が多かった。また必要な調査日数については、概して個人の習慣的な摂取量の推定を目的とした場合は若年群と男性の方が長かったが、集団内での個人の順位付けが目的の場合は、高齢群と女性の方が長かった。

結論：日本人成人において、適切な食事調査に必要な調査人数と日数を検討する際に用いられる個人内・個人間変動係数は、年齢や性、栄養素によって異なることが示された。

キーワード: 栄養素、個人内・個人間変動、年齢、性、日本人
